# Dietary Isoeugenol Supplementation Attenuates Chronic UVB-Induced Skin Photoaging and Modulates Gut Microbiota in Mice

**DOI:** 10.3390/nu16040481

**Published:** 2024-02-07

**Authors:** Ruixuan Geng, Seong-Gook Kang, Kunlun Huang, Tao Tong

**Affiliations:** 1 Key Laboratory of Precision Nutrition and Food Quality, Key Laboratory of Functional Dairy, Ministry of Education, College of Food Science and Nutritional Engineering, China Agricultural University, Beijing 100083, China; 17768128861@163.com (R.G.); foodsafety66@cau.edu.cn (K.H.); 2 Key Laboratory of Safety Assessment of Genetically Modified Organism (Food Safety), Ministry of Agriculture, Beijing 100083, China; 3 Beijing Laboratory for Food Quality and Safety, Beijing 100083, China; 4 Department of Food Engineering and Solar Salt Research Center, Mokpo National University, Muangun 58554, Republic of Korea; sgkang@mokpo.ac.kr

**Keywords:** UVB exposure, photoaging, wrinkles, collagen, isoeugenol, gut microbiota

## Abstract

Photoaging, the primary cause of skin aging damage, results from chronic ultraviolet (UV) exposure, leading to dryness and wrinkle formation. Nutritional intervention has emerged as a practical approach for preventing and addressing the effect of skin photoaging. The primary aromatic compound isolated from clove oil, isoeugenol (IE), has antibacterial, anti-inflammatory, and antioxidant qualities that work to effectively restrict skin cancer cell proliferation. This investigation delved into the advantages of IE in alleviating skin photoaging using UVB-irradiated skin fibroblasts and female SKH-1 hairless mouse models. IE alleviated UVB-induced photodamage in Hs68 dermal fibroblasts by inhibiting matrix metalloproteinase secretion and promoting extracellular matrix synthesis. In photoaged mice, dietary IE reduced wrinkles, relieved skin dryness, inhibited epidermal thickening, and prevented collagen loss. Additionally, the intestinal dysbiosis caused by prolonged UVB exposure was reduced with an IE intervention. The results of Spearman’s analysis showed a strong correlation between skin photoaging and gut microbiota. Given the almost unavoidable UVB exposure in contemporary living, this research demonstrated the efficacy of dietary IE in reversing skin photoaging, presenting a promising approach to tackle concerns related to extrinsic skin aging.

## 1. Introduction

The skin is the body’s main barrier of defense that is directly exposed to the outside world. It performs a number of vital tasks, including temperature regulation, waste excretion, stimuli detection, and protection against pathogens and injuries [1]. Skin aging is attributed to internal factors (age and genetics) and external factors (ultraviolet (UV) radiation and air pollution). Prolonged UV exposure is responsible for skin photoaging, and the concept of photoaging is historically synonymous with external skin aging, highlighting the pivotal role of UV irradiation [2]. Approximately 80% of skin aging is attributed to UV radiation exposure [3]. Photoaging is estimated to affect up to 90% of adult patients with skin phototypes I–III [4]. Skin photoaging is marked by observable signs such as deepening of wrinkles, roughness, and dryness [5]. Studies at the molecular level revealed that exposure to UVB irradiation encourages the accumulation of matrix metalloproteinases (MMP) and reduces extracellular matrix (ECM) levels in the skin [6]. In recent years, extensive studies concentrate on unraveling the molecular mechanisms of skin photoaging, involving diverse signaling pathways, molecules, and physiological processes such as mitochondrial DNA injury, oxidative stress, inflammatory response, and cellular senescence [2,3]. In addition, epidemiological studies have highlighted a significant link between various skin diseases and photoaging, including actinic elastosis, actinic keratosis, basal cell carcinoma, and melanoma [4]. Skin photoaging poses a substantial public health concern, imposing noteworthy socio-economic burdens.

Growing evidence has been presented in recent years regarding the significant influence of gut microbiota on disorders and skin health. The imbalance in gut microbial composition may increase host vulnerability and disrupt mucosal immune tolerance, subsequently affecting skin health [7,8]. Disturbances in intestinal microbiota have been linked to a number of dermatological conditions, including rosacea, psoriasis, acne, and atopic dermatitis [9]. Numerous studies have highlighted the mutual relationship between skin balance and digestive health, highlighting the important connections between the gut and skin [10]. Through metabolic processes and immunological effects, elements of the gut microbiome, such as enterobacteria and their metabolites, can affect skin conditions [11].

Various strategies are available to mitigate or prevent photoaging, encompassing physical protection measures, such as clothing and sunglasses, alongside topical treatments incorporating bioactive components and interventions in cosmetic medicine [3]. The adoption of nutritional interventions to enhance photodamaged skin has garnered growing interest, propelled by a heightened focus on improving quality of life and health awareness. There is a mounting body of evidence from both clinical and animal studies indicating that bioactive substances for instance probiotics, vitamins, oils, functional sugars, and phytochemicals could efficiently shield skin from damage caused by photoaging when incorporated into the diet [12,13].

Isoeugenol (IE), also known as 2-methoxy-4-propenylphenol, is an aromatic compound existing in the form of a transparent to yellow oily liquid, characterized by a pronounced clove fragrance. IE naturally exists in several spices like cloves, turmeric, cinnamon bark, and basil, and comprises the principal constituent in clove essential oil [14]. IE, widely utilized in the food industry, is acknowledged as an authorized substance in the National Food Safety Standard for the Use of Food Additives (GB2760-2014) [15,16]. Studies have reported that IE inhibits skin cancer cell proliferation [17] while displaying anti-inflammatory, antioxidant, and antibacterial properties [15,18]. IE demonstrates substantial potential in improving skin photoaging. This study delves into the ameliorative impact of dietary IE on chronic UVB-triggered skin photodamage and gut microbiota imbalances in mice.

## 2. Materials and Methods

### 2.1. Materials

The 98% pure IE was acquired from Sigma (St. Louis, MO, USA), and the paraformaldehyde fixative came from Servicebio Biotechnology Company (Wuhan, China). Phosphate-buffered saline (PBS, P1022) and Coomassie Brilliant Blue G250 (C8420) were provided by Solarbio Biotechnology Company in Beijing, China. Dulbecco’s Modified Eagle Medium (DMEM, D0822) was procured at Sigma in St. Louis (MO, USA). Gibco (Thermo Fisher Scientific, New York, NY, USA) supplied the penicillin-streptomycin solution (15140-122), 0.25% trypsin-ethylene diamine tetraacetic acid solution (25200-056), and fetal bovine serum (10270-106). Beyotime Biotechnology Company (Shanghai, China) provided the cell counting kit (CCK)-8 (C0037). Human hyaluronic acid (HA) ELISA kit (DHYAL0) and total MMP-1 enzyme-linked immunosorbent assay (ELISA) kit (DY008) were obtained from R&D Systems in Minneapolis (MN, USA). Procollagen type I C-peptide (PIP) ELISA kit (MK101) was supplied by Takara Bio (Shiga, Japan). Silflo silicone was sourced from Jinhongfan Trade Co. (Beijing, China), and the mouse HA ELISA kit came from CUSABIO Technology Company (Beijing, China).

### 2.2. Cell Culture and UVB Radiation

The Hs68 cell line was obtained from the American Type Culture Collection (Manassas, VA, USA). Cultured in DMEM supplemented with 1% penicillin-streptomycin solution and 10% fetal bovine serum, the cells were incubated at 37 °C in 5% CO_2_ atmospheric conditions. Medium renewal occurred every 2–3 days, and cells were passaged at 80% confluency. For subculturing, the Hs68 cells were washed with PBS, and a 0.25% trypsin/ethylenediaminetetraacetic acid (EDTA) solution was employed to detach the cells.

A UVB lamp (TL 20W/12 RS SLV/25, Philips, Amsterdam, the Netherlands) was set at a fixed distance of 30 cm from the cells to deliver UVB irradiation. An irradiance meter (Beijing Normal University Optical Instrument Factory, Beijing, China) equipped with a UVB sensor, capable of detecting UVB with a peak at 297 nm, was used to measure the irradiation intensity, measuring at 0.126 mW/cm^2^. The dosage calculations were determined using Equation (1) as follows:

(1) $$\mathrm{Irradiation}\;\mathrm{time}\;(s)=\mathrm{irradiation}\;\mathrm{dose}\;(\mathrm{mJ}/{\mathrm{cm}}^{2})/\mathrm{irradiation}\;\mathrm{intensity}\;(\mathrm{mW}/{\mathrm{cm}}^{2})$$

The application of Equation (1) enabled the determination of the necessary irradiation time to achieve the targeted UVB dose. In the UVB irradiation system for cells in this, a 40 s exposure resulted in a dose of 5 mJ/cm² for Hs68 cells. Throughout the irradiation process, the cells were maintained in PBS.

### 2.3. Cell Viability Assay

Cell viability was assessed by seeding cells at a density of 5 × 10^3^ cells/well in 96-well plates and by incubating them for 24 h, followed by a CCK8 assay. Subsequently, each well received individual treatment with either IE or the vehicle (DMSO), followed by a 24 h incubation period. The blank group consisted of only the culture medium without seeded cells. After incubation for 3 h with CCK8 solution, the optical density (OD) of the wells at 450 nm was determined utilizing a microplate reader. Equation (2) was used to calculate cell viability:

(2) $$\mathrm{Cell}\;\mathrm{viability}\;(\%)\;=\frac{\mathrm{OD}450(\mathrm{sample})\;-\;\mathrm{OD}450(\mathrm{blank})}{\mathrm{OD}450(\mathrm{control})-\mathrm{OD}450(\mathrm{blank})}\;\times \;100\;$$

### 2.4. Assessment of the HA Levels, PIP, and MMP-1 in Cells

The Hs68 cells (5 × 10^6^ cells/well) were seeded into 12-well plates and cultured for 24 h. After exposure to UVB, cells were treated with IE for 24 h, followed by cell collection. Following the manufacturers’ protocols, the MMP-1 protein content in the cellular supernatant was assessed using the human total MMP-1 ELISA kit, the quantification of collagen in the cell supernatant was conducted using the human PIP ELISA kit, and the evaluation of HA content in the cellular supernatant was performed using the human HA ELISA kit. The absorbance at 450 nm was determined by employing a microplate reader. Subsequently, the final levels of HA, PIP, and MMP-1 were normalized to the total cellular protein content.

### 2.5. Animal Experiments

The SKH-1 hairless mice have been widely employed as a model for studying skin photoaging [19]. This study followed the guidelines set forth in earlier research to examine the anti-photoaging effect of IE using SKH-1 hairless mice [6]. Animal experiments were conducted with approval from the Animal Experimental Ethical Committee and China Agricultural University Laboratory Animal Welfare (Approval No: AW30803202-4-8, Beijing, China). Female SKH-1 hairless mice (seven weeks old) were provided by Vital River Laboratories in Beijing, China, and were housed in a specific pathogen-free environment at the Animal Center (Approval No: SYXK (Jing) 2020-0052). The mice were kept in a controlled environment with a 12 h light/dark cycle at 40% to 70% relative humidity and a temperature of 22 ± 2 °C. Following a week-long acclimatization phase, twenty-four mice were divided into three groups (*n* = 8 mice per group): the control group (no UVB irradiation, fed AIN93G diet), UVB group (UVB irradiation, fed AIN93G diet), and IE group (UVB irradiation, fed AIN93G diet supplemented with 0.025% IE). The dorsal skin of the UVB and IE group mice underwent UVB radiation thrice weekly utilizing three UVB lamps (TL 40W/12 RS SLV/25, Philips, Amsterdam, The Netherlands) positioned at a constant distance of 30 cm from the irradiation target. Irradiation intensity was measured as 0.225 mW/cm^2^ using the irradiance meter. Every week, the UVB doses were progressively raised by one minimal erythemal dose (MED), with one MED equal to 100 mJ/cm^2^. The dosage was incrementally increased until it reached 3 MED, which remained consistent throughout the experiment. According to Equation (1), mice were exposed to UVB radiation three times during the first week, with each session delivering 1 MED for a duration of 7.4 min. In the second week, mice underwent UVB exposure three times, with each session providing 2 MED and lasting 14.8 min. From the 3rd to the 14th week, mice were exposed to UVB three times per week, each session comprising 3 MED and lasting 22.2 min. UVB irradiation ceased after 14 weeks. The AIN93G diet was provided to the control and UVB groups, while 0.025% IE (*w*/*w*) was added to the same diet for the IE group (Table 1). During this study, the mice were provided with free food and water access, while their food intake and body weights were consistently monitored. Following a morning fasting period of 6 h (from 8:00 a.m. to 2:00 p.m.), skin samples were obtained and immediately frozen in liquid nitrogen. The specimens were preserved at −80 °C for later examination.

### 2.6. Evaluation of Wrinkle Formation

To assess wrinkle formation, mice were anesthetized before sacrifice, and Silflo silicone was used to replicate skin wrinkles. A PRIMOS CR (Canfield Scientific, Parsippany, NJ, USA) was employed to measure various parameters, including total wrinkle volume, as well as average and maximum wrinkle depth, which was used to determine the severity of the wrinkles.

### 2.7. Evaluation of the Skin Hydration

After sacrifice and dorsal skin excision, the subcutaneous adipose was meticulously excised. Skin tissue was accurately weighed to ascertain its wet weight and then dried at 60 °C in an oven until achieving a stable weight. The calculation of skin hydration followed Equation (3):

(3) Skin hydration (%)=(wet weight−dry weight)/wet weight × 100

### 2.8. Determination of the HA Content in Skin

Mouse skin HA content was assessed by utilizing the mouse HA ELISA kit and by following the manufacturer’s instructions. The absorbance at 450 nm was measured with a microplate reader, and the ultimate HA level was adjusted based on the total protein level of the skin tissue.

### 2.9. Masson and Hematoxylin and Eosin (HE) Staining

After fixing skin samples in formalin and embedding them in paraffin, sections (5-μm) were created and subjected to staining with Masson’s trichrome and HE. An optical microscope from Olympus (Tokyo, Japan) was used for microscopic examination, and images were captured for further analysis. Image J 1.53 was used to measure the collagen density in the segments stained with Masson’s staining, and the epidermal thickness in the segments was stained with HE.

### 2.10. Fecal DNA Extraction and 16S rRNA Gene Sequencing

A fecal DNA isolation kit (FUDEAN, Beijing, China) was used for fecal DNA extraction (*n* = 6). A NanoDrop 2000 spectrophotometer (Thermo Fisher Scientific, New York, NY, USA) was employed to assess the DNA purity and concentrations in the samples. Using the 338F (5’-ACTCCTACGGGAGGCAGCAG-3’) and 806R (5’-GGACTACHVGGGTWTCTAAT-3’) primers, the16S rRNA gene sections (V3-V4) extracted were magnified. PCR process consisted of 27 cycles (30 s at 95 °C, 30 s at 55 °C, and 45 s at 72 °C). The amplicon paired-end sequencing was performed by the Majorbio Company (Shanghai, China) via PE300 chemicals and Illumina MiSeq (Shanghai, China).

### 2.11. Microbiota Data Analysis

After demultiplexing, FLASH (version 1.2.11) was employed to combine the generated sequences, while fastp (version 0.19.6) was employed for quality assessment. High-quality sequence denoising was achieved using the QIIME pipeline DADA2 plugin (version 2020.2) to derive the amplicon sequence variants (ASVs), which were classified using the SILVA 16S rRNA database (version 138) and the QIIME Naive Bayes consensus classifier (version 2020.2). Employing QIIME, we evaluated both β- and α-diversity. The Majorbio Cloud platform (www.majorbio.com) was then employed for analysis based on abund_jaccard distance to assess similarity and visualize principal coordinate analysis (PCoA). Differential bacterial taxa at the phylum and genus levels were determined using linear discriminant analysis (LDA) effect size (LEfSe) analysis with filters for LDA score > 2 and *p* < 0.05. Functional microbial community profiles were predicted using PICRUSt, and statistically significant differences were identified through STAMP (Version 2.1.3, http://kiwi.cs.dal.ca/Software/STAMP (accessed on 1 October 2023). The Majorbio Cloud Platform was utilized for data analysis, while the relationship between skin photoaging indices and the relative abundance of gut microbiota were examined via Spearman’s correlation analysis and SPSS version 17.0 (IBM, Armonk, NY, USA), while GraphPad Prism 9.4.0 (San Diego, CA, USA) was employed to create Spearman’s correlation heatmap.

### 2.12. Statistical Assessment

The outcomes are presented as mean ± SEM. The significance of variations between two groups was assessed using Student’s *t*-test, while comparisons involving more than two groups utilized one-way analysis of variance. GraphPad Prism 9.4.0 was employed for statistical assessment, with significance set at *ns* (not significant, *p* > 0.05), * *p* < 0.05, ** *p* < 0.01, *** *p* < 0.001, and **** *p* < 0.0001.

## 3. Results

### 3.1. IE Mitigates UVB Irradiation-Induced Photoaging of Hs68 Cells

**Figure 1:**
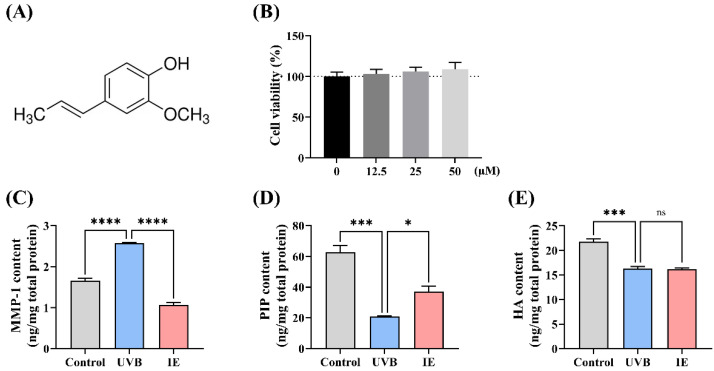
IE mitigates UVB irradiation-induced photoaging of Hs68 cells. (**A**) Chemical structure of IE. (**B**) Cell viability after 24 h incubation with 0 µM, 12.5 µM, 25 µM, and 50 µM IE. (**C**) MMP-1 contents. (**D**) PIP contents (represent the collagen contents). (**E**) HA contents. The data are presented as mean ± SEM (*n* = 3). Significant differences between the groups are indicated as * *p* < 0.05; *** *p* < 0.001; **** *p* < 0.0001; and ns, not significant (*p* > 0.05).

The impact of IE (Figure 1A) on the Hs68 cell viability was determined via a CCK8 assay. IE treatment at concentrations up to 50 µmol/L did not induce notable cytotoxicity in Hs68 cells (Figure 1B). Consequently, a 50 µmol/L IE concentration was chosen for further investigations with Hs68 cells. In accordance with our prior research, a 5 mJ/cm² UVB irradiation dose was administered to induce photoaging in Hs68 cells. UVB exposure promoted MMP-1 protein levels and decreased collagen and HA contents in Hs68 cells (Figure 1C–E). IE treatment suppressed the UVB irradiation-triggered elevation in the MMP-1 levels and significantly regained collagen levels, while it seemed not to restore the HA content (Figure 1C–E). Therefore, IE alleviated the UVB-induced photoaging of Hs68 dermal fibroblasts.

### 3.2. Dietary IE Alleviates Chronic UVB-Triggered Wrinkle Formation in Mice

**Figure 2:**
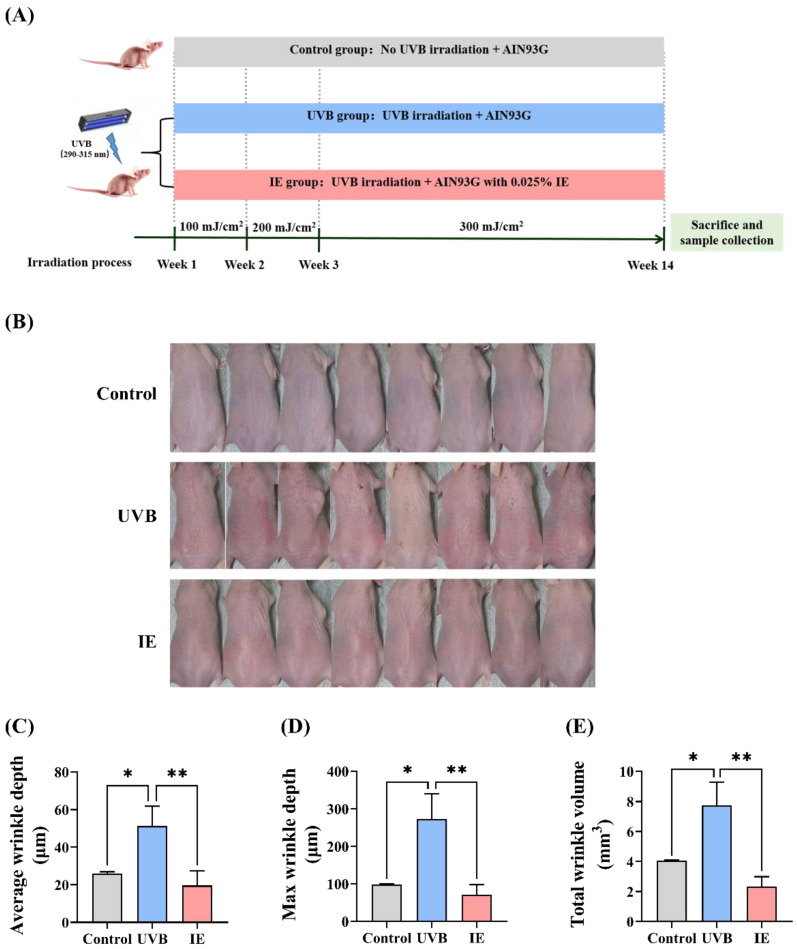
Dietary IE alleviates chronic UVB-triggered wrinkle formation in SKH-1 hairless mice. (**A**) An illustrative depiction of the experimental process. (**B**) Images depicting the dorsal skin of mice at the 14th week of the experiment. (**C**) Average wrinkle depth. (**D**) Maximum wrinkle depth. (**E**) Total wrinkle volume. The data are presented as mean ± SEM (*n* = 3–8). The significant differences between the groups are indicated as * *p* < 0.05 and ** *p* < 0.01.

In Figure 2A, the experimental timeline is presented. In contrast to unirradiated mice, those exposed to prolonged UVB exhibited the formation of skin wrinkles (Figure 2B). Dietary IE notably diminished chronic UVB irradiation-induced wrinkle formation (Figure 2B). Further evaluation of wrinkle volume and depth using silicone replicas affirmed that prolonged UVB irradiation boosted wrinkle formation. This was marked by an elevation in total wrinkle volume, average wrinkle depth, and maximum wrinkle depth (Figure 2C–E). Furthermore, the mice receiving IE supplementation showed a marked reduction in these parameters (Figure 2C–E), indicating the efficacy of IE in alleviating the negative impact of constant UVB irradiation on wrinkle formation.

### 3.3. IE Relieves Skin Dryness in Photoaged Mice

**Figure 3:**
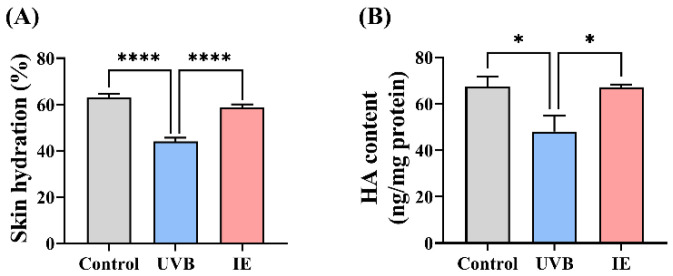
IE relieves skin dryness in photoaged mice. (**A**) Skin hydration. (**B**) HA content. The values are presented as means ± SEM (*n* = 3–8). The significant differences between the groups are indicated as * *p* < 0.05 and **** *p* < 0.0001.

Skin hydration and HA levels were examined to evaluate the capacity of IE to improve skin dryness in chronic UVB-exposed mice. Prolonged exposure to UVB irradiation reduced skin hydration and HA content, while dietary IE restored these parameters compared to the UVB group mice, consequently alleviating skin dryness (Figure 3A,B).

### 3.4. IE Inhibits Chronic UVB-Induced Epidermal Thickening in Mice

**Figure 4:**
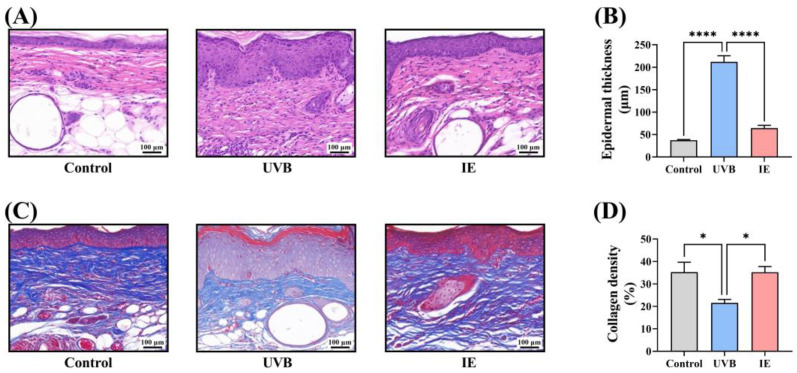
IE inhibits chronic UVB-induced epidermal thickening and collagen loss in the mouse skin. (**A**) Representative micrographs of skin sections stained with HE after 14 weeks of UVB irradiation (100×, scale bar = 100 µm). (**B**) Average epidermal thickness of the dorsal skin. (**C**) Representative micrographs of skin sections stained with Masson’s trichrome after 14 weeks of UVB irradiation (100×, scale bar = 100 µm). (**D**) Average collagen density of the dorsal skin. The data are presented as mean ± SEM (*n* = 3). The significant differences between the groups are indicated as * *p* < 0.05 and **** *p* < 0.0001.

Histological examination of skin samples after HE staining indicated that prolonged UVB irradiation led to significant epidermal thickening in the mice (Figure 4A,B). Dietary IE supplementation in the chronically UVB-exposed mice markedly reduced epidermal thickening (Figure 4A,B).

### 3.5. IE Prevents Chronic UVB-Induced Collagen Loss in Mouse Skin

Skin tissues underwent Masson’s staining to investigate the influence of IE on collagen fiber content. The collagen fiber density in the dorsal skin notably reduced following prolonged UVB exposure (Figure 4C,D). Dietary IE notably restored the collagen fiber density in the skin compared to that of mice in the UVB group (Figure 4C,D).

### 3.6. IE Induces Alterations in Both the Diversity and Composition of the Intestinal Microbiota in Mice Chronically Exposed to UVB

**Figure 5:**
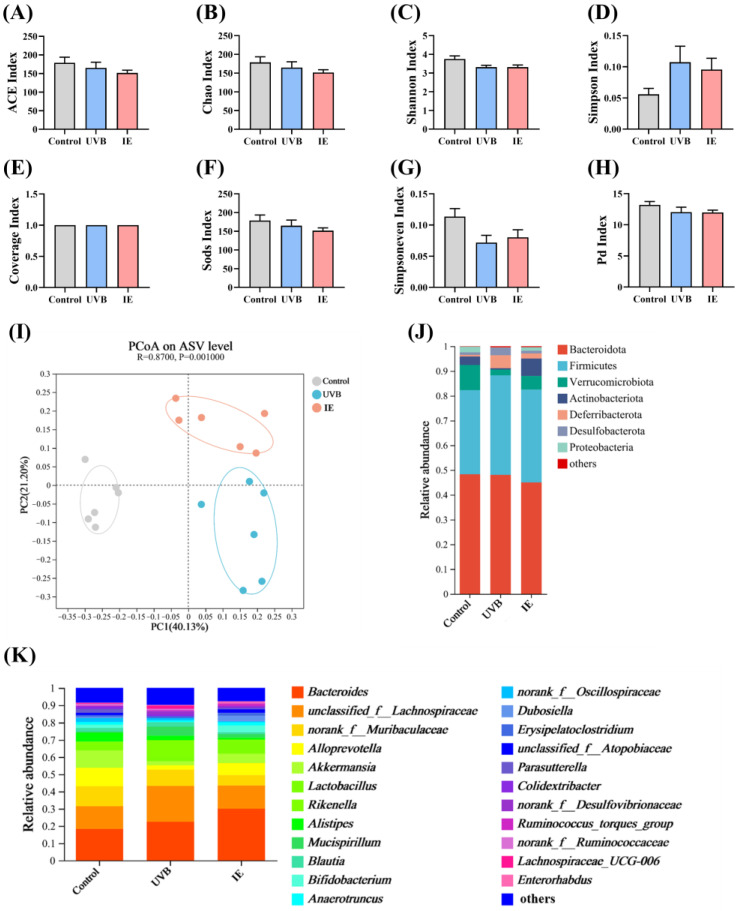
IE induces alterations in both the diversity and composition of the intestinal microbiota in mice chronically exposed to UVB. (**A**) ACE index. (**B**) Chao index. (**C**) Shannon index. (**D**) Simpson index. (**E**) Coverage index. (**F**) Sods index. (**G**) Simpsoneven index. (**H**) Pd index. (**I**) PCoA at the ASV level based on the abund_jaccard distance and assessed via analysis of similarity. (**J**) Relative abundance of the gut microbiota at the phylum level. (**K**) Relative abundance of the gut microbiota at the genus level. The data are presented as mean ± SEM (*n* = 6).

The DNA was extracted from fecal samples of the control, UVB, and IE groups, and 16S rRNA sequencing was performed to determine whether the IE intervention regulated the gut microbiota. IE supplementation’s impact on microbial communities was evaluated through ASV analysis. UVB exposure and IE intervention seemed to have no effect on α-diversity, including the Simpson, ACE, Shannon, Coverage, Chao, Sods, Simpsoneven, and Pd indices (Figure 5A–H). PCoA was performed utilizing abund_jaccard distances to evaluate β-diversity. The analysis revealed distinct segregation among the gut microbiota structures in three groups (Figure 5I), indicating substantial modifications due to prolonged UVB exposure and IE intervention. Distinct alterations in relative abundance patterns were linked to the modified gut microbial community structures at both the genus and phylum levels in the three groups (Figure 5J,K).

### 3.7. The Species Difference Analysis and Functional Prediction Analysis after IE Supplementation

**Figure 6:**
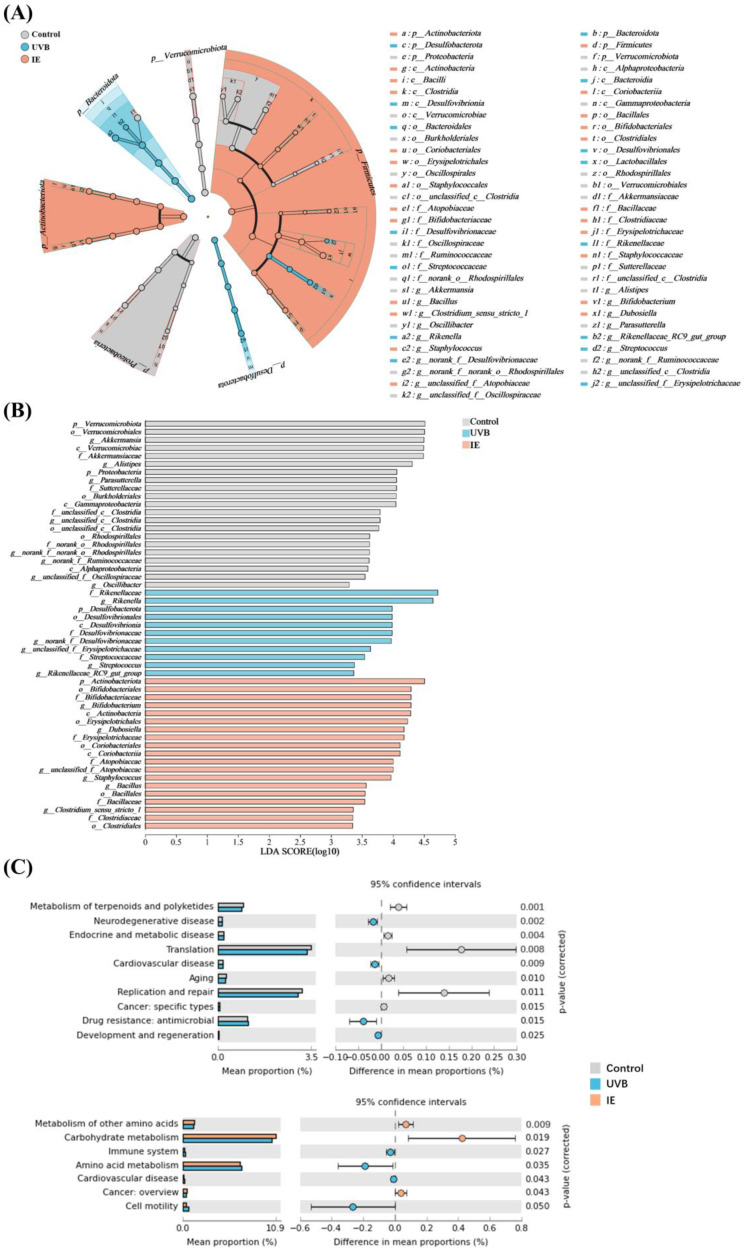
The species difference analysis and functional prediction analysis after IE supplementation. (**A**) LEfSe analysis illustrated the association of the ASVs (the concentric rings, delineating phylum, class, order, family, and genus). (**B**) A comparison between the gut microbiota of the three groups with an LDA score > 2. (**C**) The predicted function of the fecal microbiome based on the KEGG pathways. The statistical significance of the differences was determined using a *t*-test (*p* < 0.05) in STAMP. *n* = 6.

LEfSe analyses were performed to detect the distinctly abundant bacterial taxa in the three groups. At the ASV level, the LEfSe analysis was performed using a two-point scoring threshold. The distinctly abundant bacterial taxa of the control group mice were *Verrucomicrobiota* and *Proteobacteria* at the phylum level; *Verrucomicrobia*, *Gammaproteobacteria*, and *Alphaproteobacteria* at the class level; *Verrucomicrobiales*, *Burkholderiales*, *unclassified_c__Clostridia*, and *Rhodospirillales* at the order level; *Akkermansia*, *Alistipes*, *Parasutterella*, *Parasutterella*, *unclassified_c__Clostridia*, *norank_f__norank_o__Rhodospirillales*, *norank_f__Ruminococcaceae*, *unclassified_f__Oscillospiraceae*, and *g__Oscillibacter* at the genus level; and *Akkermansiaceae*, *Sutterellaceae*, *unclassified_c__Clostridia*, and *norank_o__Rhodospirillales* at the species level (Figure 6A,B). The distinctly abundant bacterial taxa of the UVB group mice were *Desulfobacterota* at the phylum level; *Desulfovibrionia* at the class level; *Desulfovibrionales* at the order level; *Rikenellaceae*, *Desulfovibrionaceae*, and *Streptococcaceae* at the family level; and *Rikenella*, *norank_f__Desulfovibrionaceae*, *unclassified_f__Erysipelotrichaceae*, *Streptococcus*, and *Rikenellaceae_RC9_gut_group* at the genus level (Figure 6A,B). The distinctly abundant bacterial taxa of the IE group mice were *Actinobacteriota* at the phylum level; *Actinobacteria* and *Coriobacteriia* at the class level; *Bifidobacteriales*, *Erysipelotrichales*, *Coriobacteriales*, *Bacillales*, and *Clostridiales* at the order level; *Bifidobacteriaceae*, *Erysipelotrichaceae*, *Atopobiaceae*, *Bacillaceae*, and *Clostridiaceae* at the family level; and *Bifidobacterium*, *Dubosiella*, *unclassified_f__Atopobiaceae*, *Staphylococcus*, *Bacillus*, and *Clostridium_sensu_stricto_1* at the genus level (Figure 6A,B). To elucidate the changes in microbial community functionality following prolonged UVB irradiation and the IE intervention, the KEGG database was used for the 16S rRNA gene catalog annotation. Chronic UVB exposure upregulated neurodegenerative disease, cardiovascular disease, antimicrobial drug resistance, and the development and regeneration while downregulating terpenoids and polyketide metabolism, endocrine and metabolic disease, translation, aging, replication and repair, and cancer—specific types (Figure 6C). The IE intervention upregulated the metabolism of other amino acids, carbohydrate metabolism, and cancer (overview), and downregulated immune disease, amino acid metabolism, cardiovascular disease, and cell motility (Figure 6C).

### 3.8. The Correlation Assessment between the Relative Abundance of Gut Microbiota and Skin Photoaging Indices

**Figure 7:**
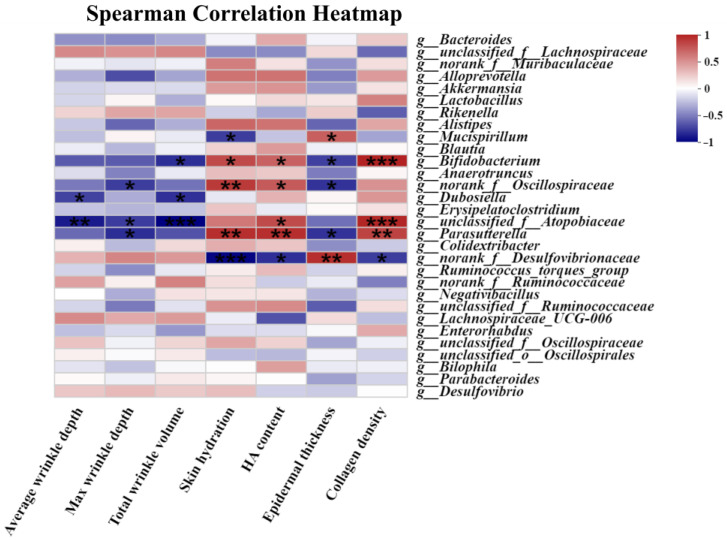
The correlation assessment between the relative abundance of gut microbiota and skin photoaging indices. Blue denotes a negative correlation, and red indicates a positive correlation. The significant differences between the groups are indicated as * *p* < 0.05, ** *p* < 0.01, and *** *p* < 0.001. *n* = 3.

The association between the skin photoaging indices and the relative abundance at the genus level of gut microbiota (top 30) was analyzed. Figure 7 demonstrated the noteworthy correlation between the skin photoaging indices (total wrinkle volume, average and maximum wrinkle depths, skin hydration, HA content, epidermal thickness, and collagen density) and the relative abundance at the genus level of gut microbiota after correlation analysis. For instance, the relative *Mucispirillum* abundance changes were positively related to epidermal thickness and negatively related to skin hydration. The relative *Bifidobacterium* abundance changes was inversely connected with total wrinkle volume and epidermal thickening and positively related to skin hydration, HA content, and collagen density. The alterations in the relative *Dubosiella* abundance were negatively related to total wrinkle volume and the average wrinkle depth. The changes in the relative *Parasutterella* abundance were positively related with skin hydration, HA content, and collagen density, and were negatively related with the maximum wrinkle depth and epidermal thickness.

## 4. Discussion

Moderate UV radiation levels have a number of health benefits, including the removal of microorganisms; the regulation of the nervous, endocrine, digestive, and respiratory systems; the strengthening of the immune system; and the facilitation of vitamin D synthesis [20]. Nonetheless, extended exposure to low-concentration UV radiation or abrupt spikes in UV radiation can be harmful to the skin, eyes, and immune system [21]. The detrimental effects of ongoing UV radiation exposure on the skin and internal organs have been thoroughly investigated and are well-established [22]. The utilization of dietary interventions has become increasingly popular as a common strategy to address the outcomes of extrinsic skin aging induced by UV radiation [3]. Our research underscores the efficacy of incorporating dietary IE to counter the risks linked to skin photoaging caused by prolonged UVB radiation exposure.

IE showed a beneficial effect on restoring UVB-induced decreases in collagen and HA content both in vivo and in vitro. In the skin, fibroblasts represent the major dermis cell types, producing ECM components, including collagen and HA [5]. Photoaged fibroblasts are the crucial driving factor in the skin’s photoaging process [23]. Photodamage of fibroblasts reduces ECM, ultimately causing undesirable effects such as skin dryness and wrinkle formation [2].

The Food and Drug Administration has endorsed the use of IE as an additive. A repeated dose toxicity examination confirmed the no-observed-adverse-effect level for IE in mice at 37.5 mg/kg body weight/day [24]. The benchmark dose-based approach revealed a point of departure of 8 mg/kg b.w./day for IE in mice and the results of this study confirmed a low risk of the estimated daily exposure levels of IE [25]. The dosage administered to the mice in this research equated to 0.12 g/day for an adult woman (weighing an average of 59 kg) [26]. IE absorption and metabolism were evaluated in a previous study in which mice were administered a dose of 140 mg/kg of IE via gavage, with no reported adverse clinical signs [27]. The IE dosage (25 mg/kg) administered to mice in this investigation was below the no-observed-adverse-effect level and lower than the dosages employed in other studies. Throughout the course of this experiment, no adverse reactions were detected.

Chronic UVB skin exposure induces gut microbiota disorders in mice. Regarding the diversity changes in the gut microbiota after UV exposure, Ghaly et al. reported no significant alteration in the fecal microbiota α- and β-diversity in female C57BL/6 mice after 4 weeks of UVB exposure, based on operational taxonomic unit analysis [28]. Our prior investigation indicated that, after 14 weeks of chronic UVB exposure, α-diversity did not change significantly, while β-diversity was substantially altered, based on ASV analysis [22]. This study demonstrated that 14 weeks of UVB exposure did not notably change the α-diversity of the intestinal microbiota, while β-diversity changed substantially in the female SKH-1 hairless mice. Concerning the characteristic gut bacteria after UV exposure, Ghaly et al. revealed that the relative abundance of *Coprococcus* and *Mucispirillum* was notably increased, while that of *Bacteroidales* was significantly reduced [28]. Our prior investigation indicated that the relative abundance of *Lachnospiraceae_NK4A136_group*, *unclassified_f__Ruminococcaceae*, and *Lactobacillus* increased significantly at the genus level, while that of *Alloprevotella*, *Tuzzerella*, *Roseburia*, and *norank_f__norank_o__Clostridia_vadinBB60_group* decreased considerably after chronic UVB exposure, based on LEfSe analysis [22]. The LEfSe analysis in this study identified several characteristic gut bacteria at the genus level after UVB irradiation, although they were not consistent with the intestinal strains found in the studies described above [22,28]. These inconsistent changes in diversity and characteristic bacteria of gut microbiota may result from variances in mouse strain, gender, diet, housing conditions, UVB exposure dosage, duration of exposure, and analytical approaches.

Research has indicated a robust correlation between the functional, compositional, and structural characteristics of the gut microbiota and the aging process of the skin [3,29]. Human studies suggest a transition from the Firmicutes phylum to an elevated abundance of the Bacteroidetes phylum with increasing age [30]. As mice age, there is a noticeable decrease in favorable anti-inflammatory microbial strains—for example, Bifidobacteria and Faecalibacterium—in their intestines [31]. This study showed that photoaged mice had altered gut microbiota. Notably, the dietary IE intervention significantly reduced gut microbiota imbalances and skin photodamage, indicating a possible link between the IE’s ability to reduce skin photoaging and decrease disruptions in the intestinal microbial environment.

The characteristic gut bacteria at the genus level in the UVB group identified via LEfSe analysis were associated with skin diseases. For instance, studies have shown that *Streptococcus*, one of the predominant genera in the gut lumen [32], was significantly elevated in the feces of psoriasis patients and mice [33,34]. The relative abundance of *Rikenella* was considerably elevated in the feces of psoriatic mice [35]. Mice with cutaneous acne induced by oleic acid showed a notable upregulation in the relative abundance of *Rikenellaceae_RC9_gut_group*, which was positively correlated with inflammatory factor levels [36]. The characteristic gut bacteria at the genus level of the IE group identified via LEfSe analysis appeared to alleviate skin disorders. *Bifidobacterium*, the most common probiotic, was demonstrated to improve skin barrier dysfunction, delay skin aging, and alleviate atopic dermatitis by regulating gut–skin axis homeostasis [37,38]. The relative abundance of *Dubosiella* decreased substantially in the feces of treated atopic dermatitis mice [39]. We showed that the relative abundance of *Dubosiella* was negatively correlated with wrinkle-related indicators. The relative abundance of *Clostridium_sensu_stricto_1* was significantly lower in the feces of adults with atopic dermatitis compared to individuals without this condition [40]. However, research on the role of these characteristic gut bacteria during the skin’s photoaging process remains lacking and warrants further exploration.

In this study, we employed female SKH-1 hairless mice to investigate the preventive effects of IE on photodamage and gut microbiota disorders. Studies have emphasized the pivotal role of gender in the initiation of various diseases, highlighting differences in responses between males and females to uniform stimuli, such as UV irradiation [41]. Previous studies have shown that male SKH-1 hairless mice display a relatively delayed response to the photoimmune impact induced by sole UV irradiation, showing lower expression levels of inflammatory factors compared to their female counterparts [42]. However, contradictory research points to the possibility that male Kunming mice may be more susceptible than females to long-term UV radiation, which could significantly increase inflammatory skin damage and oxidative stress [43]. One area of our future research focuses on the different ways that mice respond to dietary interventions and photodamage to the skin based on their gender.

## 5. Conclusions

Overall, IE effectively mitigates skin photoaging in vitro and in vivo. IE demonstrates the ability to alleviate UVB irradiation-induced photodamage in dermal Hs68 cells, inhibiting MMP secretion and promoting ECM synthesis. Dietary IE mitigates wrinkle formation, relieves skin dryness, inhibits epidermal thickening, and prevents collagen loss in the skin. The 16S rRNA sequencing analysis indicates that IE efficiently alleviates chronic UVB-induced intestinal dysbiosis. Additional correlation analysis shows an intimate correlation between the parameters related to skin photoaging and gut microbiota. The results underscore the encouraging prospect of IE as a feasible intervention for the prevention of skin photodamage, presenting new avenues for alternative strategies to revert extrinsic skin aging.

## Figures and Tables

**Table 1:** Dietary composition (g/kg) administered to mice.

Ingredients	Control and UVB Groups (AIN93G Diet, g/kg)	IE Group (AIN93G Diet Supplemented with 0.025% IE, g/kg)
Corn starch	397.486	397.236
Casein	200	200
Maltodextrin	132	132
Sucrose	100	100
Soybean oil	70	70
Cellulose	50	50
Mineral mix 1	35	35
Vitamin mix 2	10	10
L-Cystine	3	3
Choline bitartrate	2.5	2.5
Tert-Butylhydroquinone	0.014	0.014
IE	-	0.25

1 Mineral mix: AIN93G–Mineral mix. 2 Vitamin mix: AIN93G–Vitamin mix.

## Data Availability

The data that support the findings of this study are available from the corresponding author upon reasonable request.
